# Stacked printed MoS_2_ and Ag electrodes using electrohydrodynamic jet printing for thin-film transistors

**DOI:** 10.1038/s41598-022-27072-3

**Published:** 2022-12-28

**Authors:** Thi Thu Thuy Can, Woon-Seop Choi

**Affiliations:** 1 School of Electronics and Display Engineering, Hoseo University, Asan, 31499 Korea; 2 Present Address: Faculty of Physics, Hanoi National University of Education, Hanoi, Vietnam

**Keywords:** Materials science, Nanoscience and technology

## Abstract

Transition metal dichalcogenide-based thin-film transistors (TFTs) have drawn intense research attention, but they suffer from high cost of materials and complex methods. Directly printed transistors have been in the limelight due to low cost and an environmentally friendly technique. An electrohydrodynamic (EHD) jet printing technique was employed to pattern both MoS_2_ active layer and Ag source and drain (S/D) electrodes. Printed MoS_2_ lines were patterned on a silicon wafer using a precursor solution and simple annealing, and the patterns were transferred on other SiO_2_ substrates for TFT fabrication. On top of the patterned MoS_2_, Ag paste was also patterned for S/D electrodes using EHD jet printing. The printed TFTs had a high on–off current ratio exceeding 10^5^, low subthreshold slope, and better hysteresis behavior after transferring MoS_2_ patterns. This result could be important for practical TFT applications and could be extended to other 2D materials.

## Introduction

As representative 2D materials, the transition metal dichalcogenide (TMD) group has virtually taken the place of graphene due to a novel feature of controllable band structure. It has excellent semiconducting properties with a tunable bandgap from a single layer (1.83 eV) to multiple layers (1.2 eV). Stacked-layer MoS_2_ is one of the most appropriate alternatives to graphene for the fabrication of TFT devices. Furthermore, multi-layer MoS_2_ is more highly evaluated when compared to mono-layer MoS_2_ regarding improvement of the current drive of TFTs because of a large density of states, which creates multiple conducting channels due to field effects^1,2^.

Scientists employ mechanical^3^ or chemical exfoliation^4^ to create good quality film by taking advantage of weak van der Waals forces of interlayer bonding. However, they face limitations in terms of micron size, shape, and thickness control. Concerning these problems, a chemical vapor deposition (CVD) method was proposed for the sulfurization of metal thin films or vapor phase reaction of metal oxides with a chalcogen precursor, which was an effective way to achieve large-area growth. However, the drawbacks of this method for clean and reproducible growth MoS_2_ were uncontrolled S or MoO_3_ diffusion flow^5^, a thermal evaporation requirement for the smoothness of pre-deposited MoO_3_^6^ and a multi-step process^7^. Alternatively, vacuum techniques such as atomic layer deposition (ALD) can obtain exactly the desired number layers, but they were costly^8^.

To overcome these disadvantages, solution-based methods such as spin-coating are simpler and save more time and cost than the ones mentioned. In our previous work, we investigated a new precursor solution for MoS_2_ using ammonium tetrathiomolybdate^9^. The method of making the MoS_2_ layer was emphasized to be CVD-free without sulfurization using a spin-coating technique. Nevertheless, it was our first try at synthesizing MoS_2_ and applying it on thin film transistors, and high performance devices were unsuccessfully obtained from spin-coated MoS_2_.

Jet printing techniques were investigated as an alternative for spin-coating, which was unsuitable for the requirements of micro-scale, pattern shape, and material usage. Some works proposed inkjet printing of several 2D materials such as graphene, hexagonal boron nitride h-BN and MoS_2_ for field effect transistors (FETs)^10^. However, the grown films still possessed lower resolution and a rough surface (~ 100 nm), and only low-viscosity ink can be applied in inkjet printing.

Electrohydrodynamic (EHD) jet printing is a technique that uses electric fields to yield fluid flows for delivering solution/paste materials to a target substrate. EHD printing can create smooth areas or patterns with a large range of material viscosity, even with low-viscosity solutions or high-viscosity pastes, which are better by far than inkjet printing for this concern. Moreover, the merit of EHD jet printing is that MoS_2_ TFTs can be patterned simply without using any shadow masks compared to other methods such as E-beam or thermal evaporation. This merit facilitates the practical development of a variety of functional devices. Recently, direct-patterned source and drain (S/D) electrodes have been reported in oxide and organic thin film teansistor applications using this printing techinque^11,12^.

**Figure 1:**
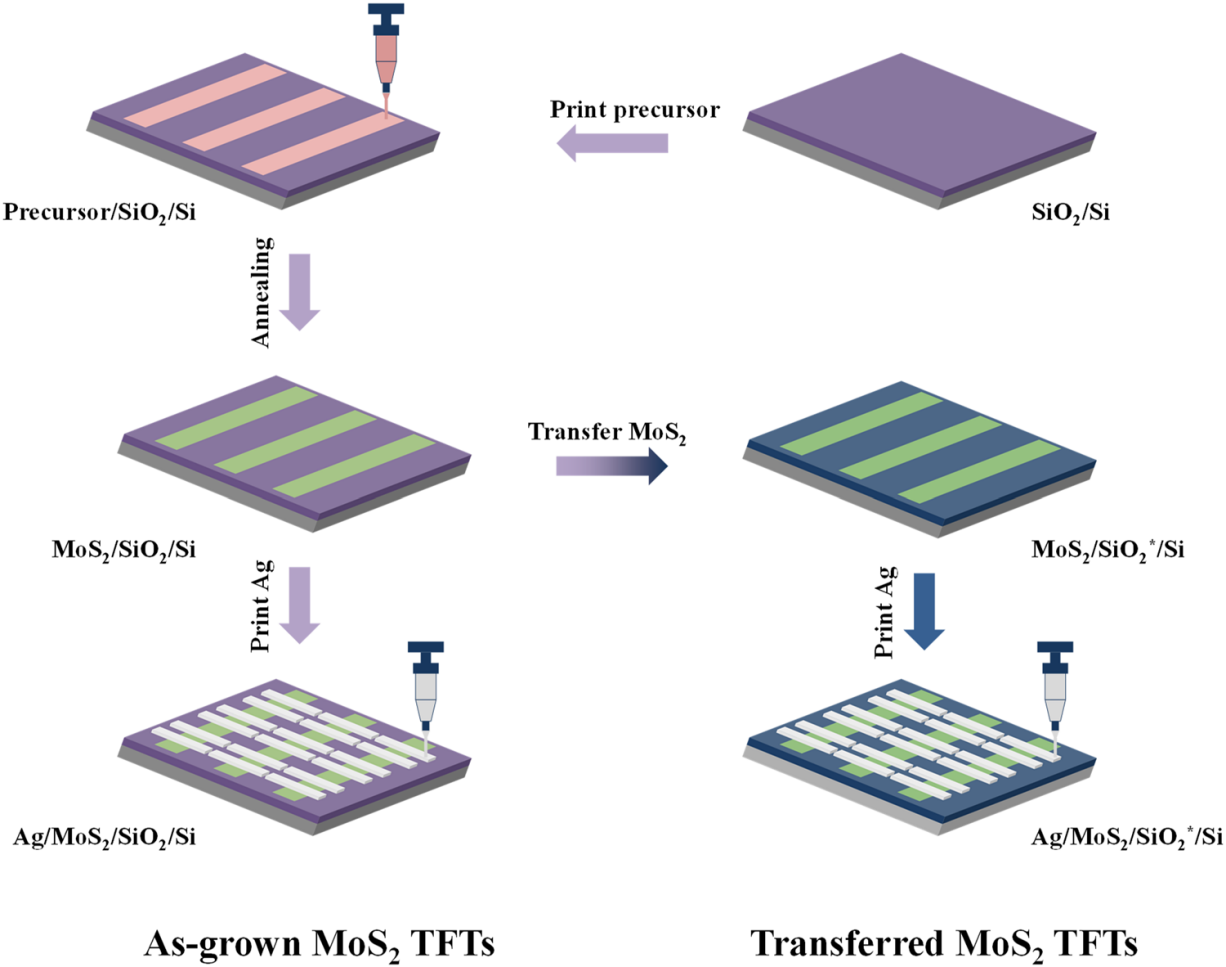
Printing and transferring procedure employed to obtain MoS_2_ pattern on 300 nm-thick-SiO_2_ and another substrate and fabrication process of MoS_2_ TFTs. SiO_2_^*^ denotes another clean 300 nm-thick-SiO_2_ used to transfer MoS_2_.

In this work, MoS_2_ thin-film transistors were fabricated with a unique combination of simple-strategy patterning MoS_2_ from precursor solution and S/D electrodes using an EHD jet printing method, as shown in Fig. 1. For designing printed electronics, silver nanoparticle paste was selected for the S/D due to a host of advantages, such as printable material, affordable price, high conductivity, resistance to oxidization, good adhesion to oxide, and matching work function with the conduction band of MoS_2_. The TFT performance of multilayer MoS_2_ transistors was evaluated before and after transfer on the same type of SiO_2_/Si substrate. Interestingly, transferred MoS_2_ TFTs showed better electrical characteristics compared to as-grown MoS_2_ TFTs. The hysteresis behavior was also characterized, and we obtained a remarkable decrease of the hysteresis phenomenon after transferring.

## Results and discussion

### EHD jet patterning MoS_2_ and Ag lines

**Figure 2:**
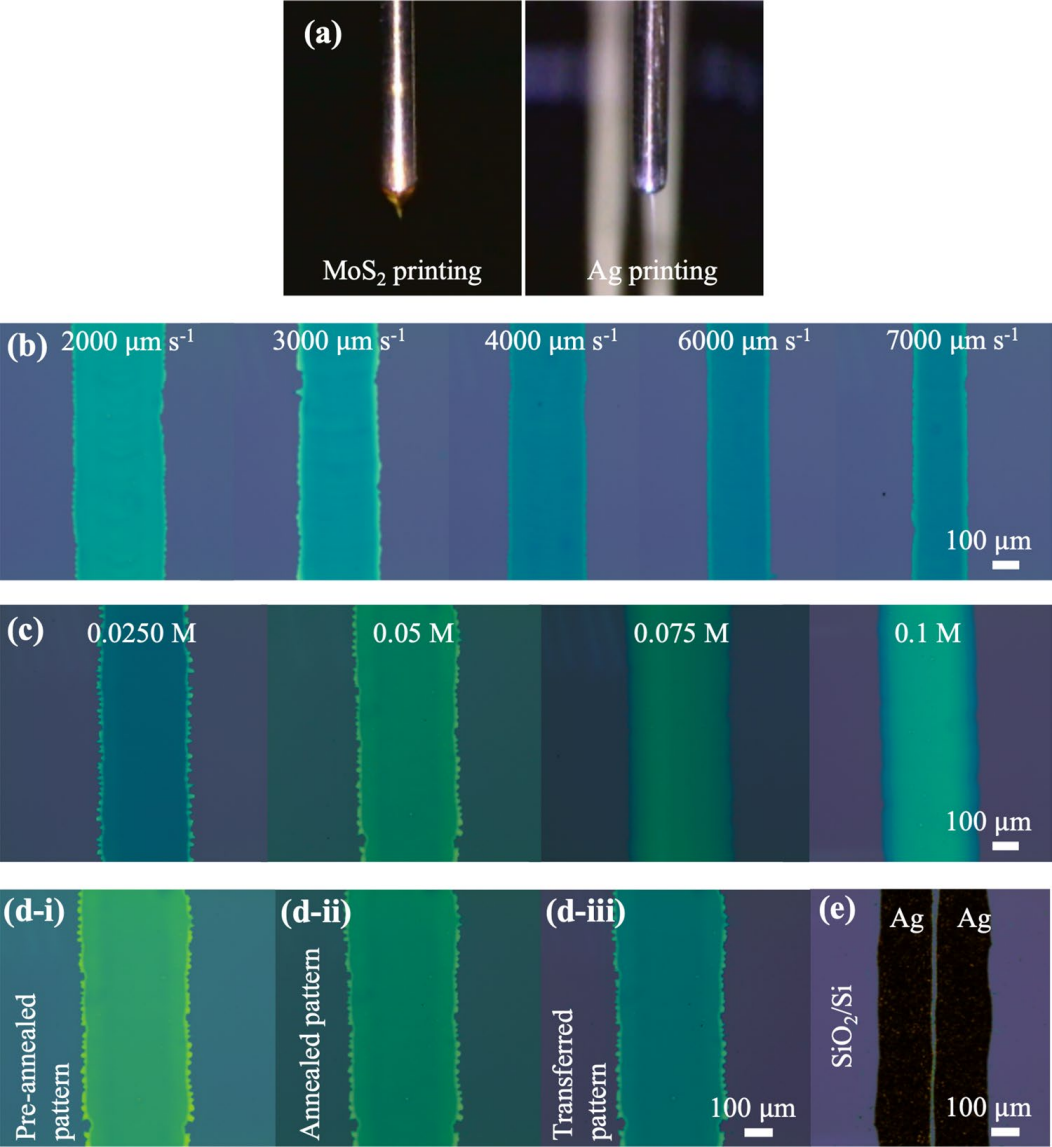
Taylor cone-jet mode used to print (**a**) MoS_2_ and Ag lines. (**b**) Optical images of MoS_2_ lines printed at various stage speeds from 2000 to 7000 µm s^−1^. (**c**) Optical images of MoS_2_ printed from different concentrations from 0.025 M to 0.1 M at the same stage speed of 3000 µm s^−1^. (**d**) Optical images of 0.05 M concentration-based MoS_2_ patterns: (i) pre-annealed and (ii) annealed on SiO_2_/Si, (iii) transferred on another SiO_2_/Si. (**e**) Optical microscope image of Ag pattern lines on SiO_2_/Si for testing.

Optimizing printing parameters is of importance to get a desirable jetting mode. In this work, Taylor cone-jet mode was used for both semiconductor and S/D electrodes in linear shape. Precursor fluids flowing through 100-µm inner-diameter capillary nozzles in the EHD jet printer resulted in 100-µm scale (Fig. 2a). A good combination of printing parameters is important to obtain Taylor cone jet mode for patterning linear MoS_2_ and Ag lines as discussed previously the effect of printing parameters on pattern quality^13,14^.

For printing MoS_2_ patterns, the parameters were a 1.8-mm tip height, 50 °C substrate temperature, 0.0032 µ L s^−1^ flow rate, and 1.8-kV applied voltage for the (NH_4_)_2_MoS_4_ precursor. The stage speed was then adapted to obtain uniformity and the required size of patterns. To investigate the substrate velocity’s effect on pattern neatness, the movement speed was varied from 2000 to 7000 µ m s^−1^ while maintaining all other parameters during the printing process. A high-speed process with a lower contact time of the jetting solution leads to smaller line width, as shown in Fig. 2b.

0.025, 0.05, 0.075, and 0.1 M solutions of (NH_4_)_2_MoS_4_ were prepared at the same stage speed of 3000 µm s^−1^ to check a printing range of precursor concentrations. Optical microscope images of corresponding patterns were obtained after drying at 150 °C, as shown in Fig. 2c. Changing color of the pattern was observed from yellow (0.1 M) to green-like as the concentration was diluted from 0.1 M to lower concentrations. The shift in colors suggested that the (NH_4_)_2_MoS_4_ thickness increased with the precursor concentration. From the optical images of printed (NH_4_)_2_MoS_4_ lines, the printability of (NH_4_)_2_MoS_4_ solution by EHD jet printing was confirmed.

Figure 2d shows optical images of the same MoS_2_ patterns printed on SiO_2_/Si from 0.05 M precursor concentration before annealing, after annealing, and after transferring them onto another SiO_2_ substrate. No wrinkles or contaminants of MoS_2_ thin films were observed after annealing and transferring thin films compared to pre-annealed MoS_2_. This demonstrated that undamaged MoS_2_ thin films were successfully obtained, and the nature of the MoS_2_ remained.

A drawback of this printing technique is that the significantly charged droplets give rise to the solution/paste shooting out in all directions on the substrate. This could be reduced by modifying the original solution/paste and relevant printing parameters. To create a clear Ag pattern on a MoS_2_ pattern (Fig. 2e), tiny amounts of PGMEA and Silveray were added to the original silver nanoparticles. The PGMEA is used to improve droplet formation and enhances the printed feature resolution^13^. Due to different properties such as the low viscosity of (NH_4_)_2_MoS_4_ solution and high viscosity of Ag paste, the system of operating parameters was different. Those were well-discussed in our previous papers regarding patterning Ag and MoS_2_ linear lines^13,14^. After optimization of the operating parameters, stacked Ag S/D on MoS_2_ patterns were obtained using EHD jet printing.

### Printed MoS_2_ and Ag features

**Figure 3:**
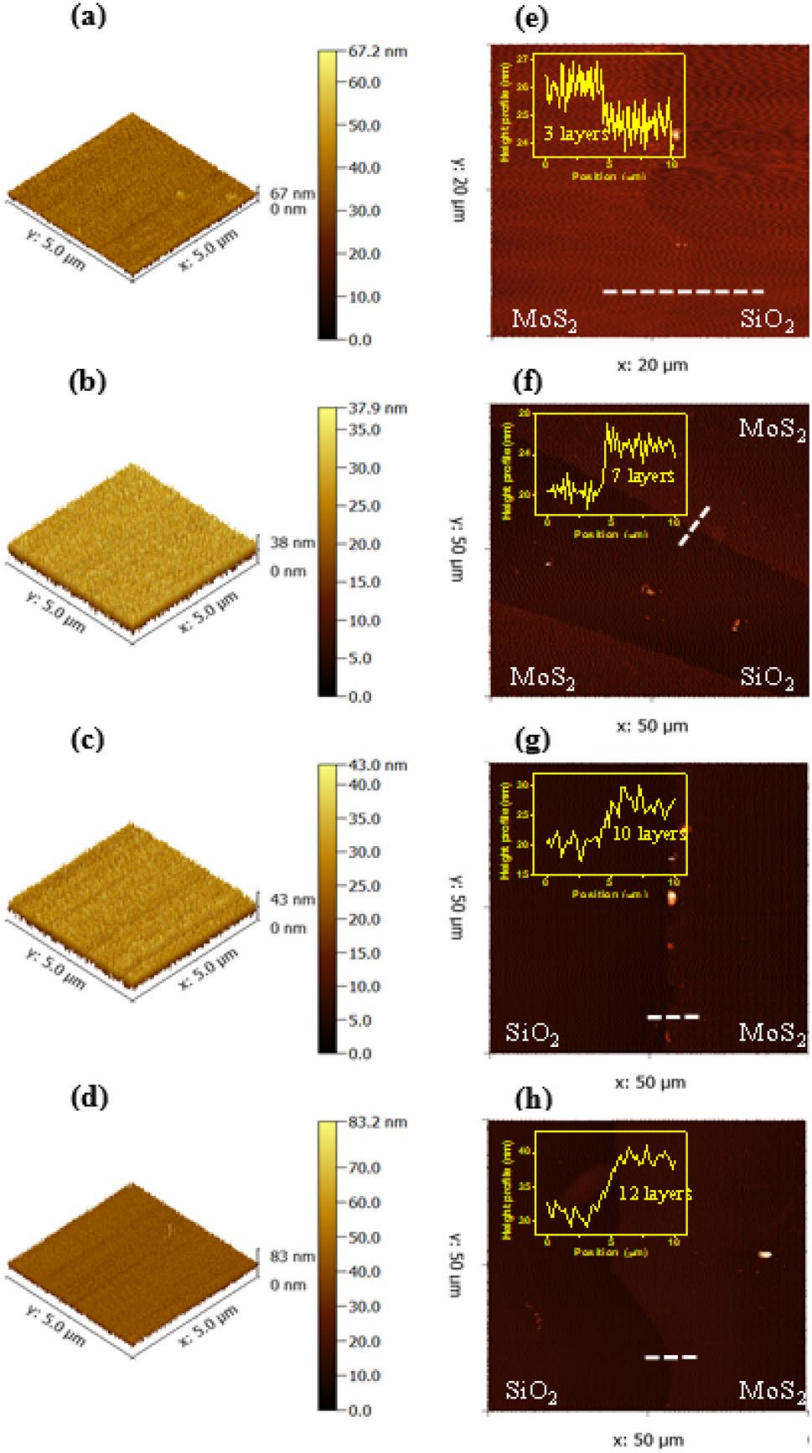
AFM images at surface of printed MoS_2_ on SiO_2_ and at the MoS_2_–SiO_2_ boundary with height profile taken from the corresponding white dash line: (**a**, **e**) 0.025 M, (**b**, **f**) 0.05 M, (**c**, **g**) 0.075 M and (**d**, **h**) 0.1 M.

Controlling the smoothness and thickness of MoS_2_ plays a crucial role because the number of layers considerably affects the optical and electrical properties of MoS_2_. Patterns printed from four concentrations were measured for their morphology and height profile using a contact-mode AFM. The root mean square (RMS) roughness values were 0.20–0.25 nm for the MoS_2_ patterns (Fig. 3a–d). These values revealed reliable uniformity and smoothness of the MoS_2_ surfaces, which is also important for upper layer deposition.

Due to the theoretical thickness of the MoS_2_ monolayer (0.65 nm), the EHD jet printing technique demonstrates its potential to fabricate few-layer films. The thickness of patterns was derived from MoS_2_ transferred on other SiO_2_/Si substrates. The results show the MoS_2_ lines produced from concentrations of 0.025–0.1 M exhibited three to twelve layers (Fig. 3e–h). Thus, the thickness can be controlled by modifying the solution concentration in the EHD jet printing.

The reliability of making uniform patterns from EHD jet printing could be understood. A computer-integrated EHD jet printer can make fine patterns with high resolution and uniformity. Therefore, with the same deposition parameters of both printing process and physics solution properties, the same MoS_2_ layer can straightforwardly achieved after growth procedure. Then good uniformity can be obtained from this run-to-run process.

Raman spectroscopy was utilized to predict the thickness of the MoS_2_ samples. Two typical concentrations of 0.025 and 0.05 M were selected. Fig. S1a,b in the Supplementary Information (S.I.) illustrate Raman spectra collected from three arbitrary positions for each sample of MoS_2_ patterns. The two main peaks were ${\text{E}}_{{2{\text{g}}}}^{1}$ and ${\text{A}}_{{1{\text{g}}}}$ peaks correspond to in-plane Mo and S atoms and out-of-plane vibrations of S atoms in the Raman shift range of 360–430 cm^−1^ for general MoS_2_. The overlap of individual Raman spectra for each concentration demonstrated the uniformity of the printed MoS_2_ growth. There is a tight relationship of the frequency difference $(\Delta \kappa )$ between the ${\text{A}}_{{1{\text{g}}}}$ and ${\text{E}}_{{2{\text{g}}}}^{1}$ peaks and the thickness of MoS_2_. The obtained $\Delta \kappa$ value of 23.3 and 24.9 suggest that three and five layers could be obtained from 0.025 and 0.05 M samples^15^.

**Figure 4:**
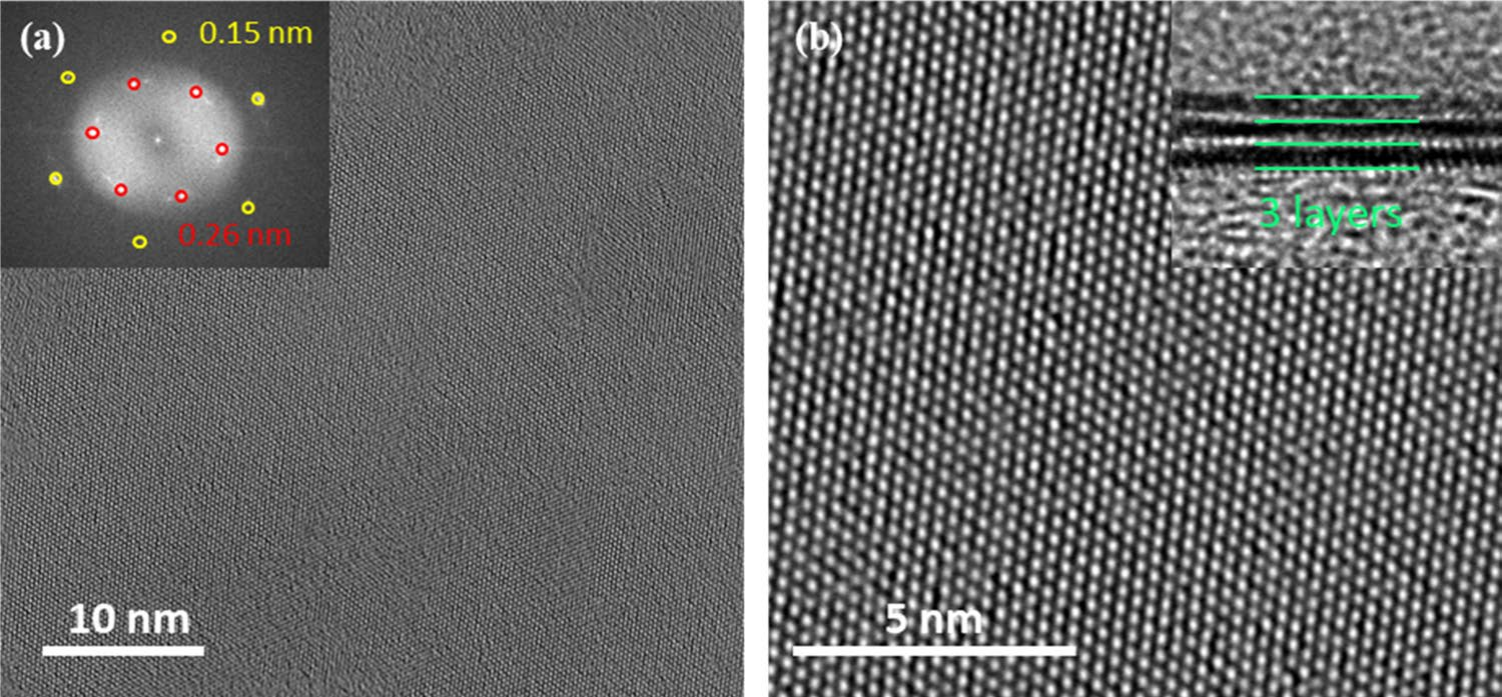
(**a**) Plan-view TEM image of printed MoS_2_ prepared from 0.025 M on SiO_2_/Si. The inset is the FFT pattern. (**b**) High-resolution TEM image. The inset is a cross-section TEM image of 0.025 M solution-based MoS_2_.

A transmission electron microscope was utilized to probe the microstructure surface of printed MoS_2_. Figure 4 shows the plan-view TEM image at low resolution showing uniform color, which means an even surface of printed 0.025 M MoS_2_. The inset of Fig. 4a shows the fast Fourier transform (FFT) pattern. For more details, the red and yellow numbers denote that the corresponding FFT pattern reveals a hexagonal lattice structure with lattice spacing of 0.26 and 0.15 nm, which are assigned to the (100) and (110) directions. The high-resolution TEM (HRTEM) image in Fig. 4b reveals the periodic atom arrangement of the printed MoS_2_. This HRTEM shows the highly polycrystalline hexagonal lattice structure of printed MoS_2_. The parallel lines correspond to stacked tri-layer MoS_2_, as shown in the cross-sectional TEM image (the inset of Fig. 4b).

As shown by the SEM measurement (Fig. S2a,b in the S.I.), a uniform surface of silver after drying was observed in the form of connected nanoparticles at different scales of printed Ag on MoS_2_. A cross-sectional SEM image (Fig. S2c) and its inset show the morphology and thickness of each layer. 2 µm-thick Ag was obtained from EHD jet printing. With the same printing parameters, the width of Ag lines on MoS_2_ was similar to that of the line printed on SiO_2_/Si in Fig. 2b. To easily measure the TFTs’ electrical properties, Ag patterns for the S/D were designed with approximately 2  µm thickness and 200  µm width.

### TFT application

**Figure 5:**
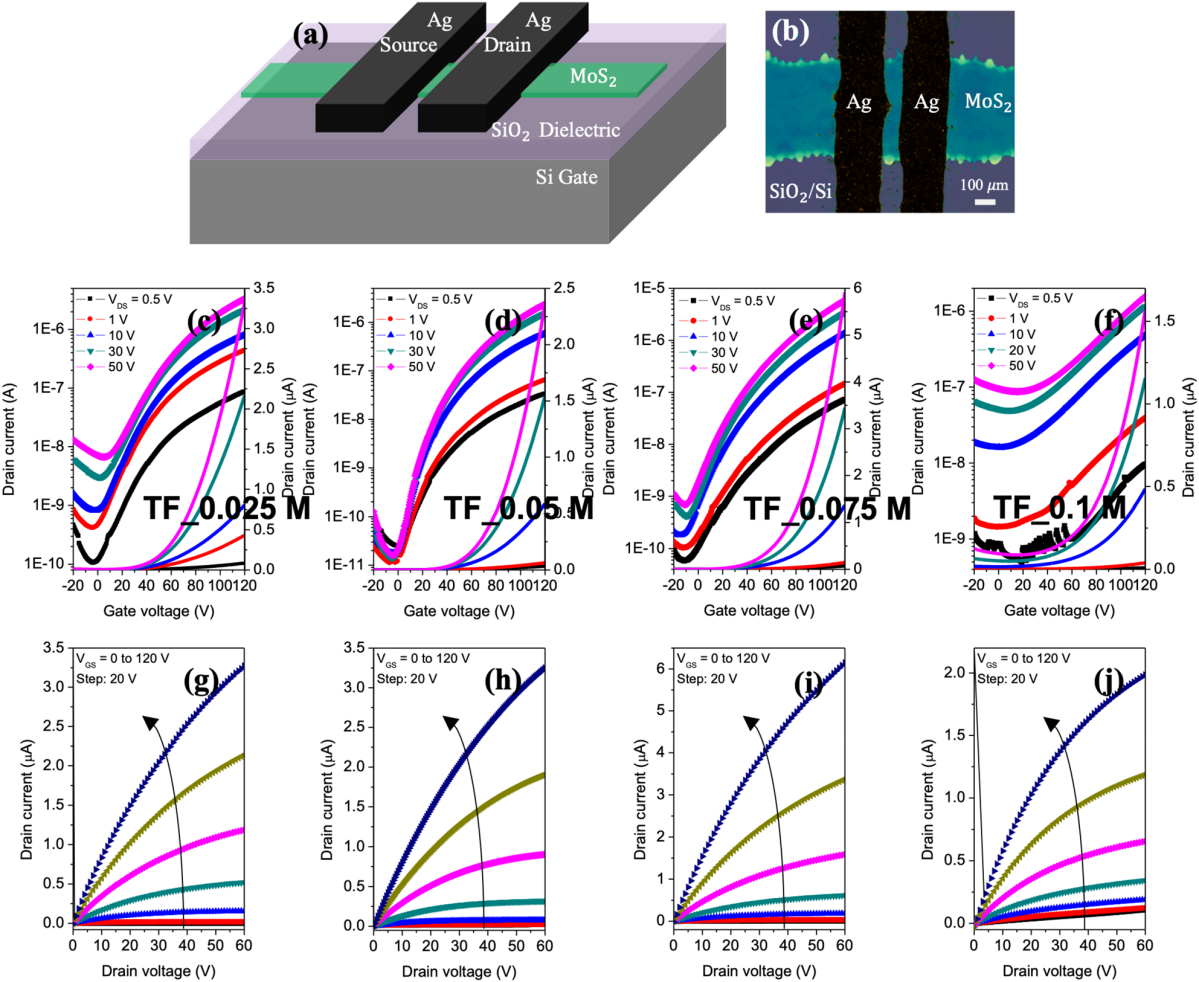
(**a**) Diagram of transferred MoS_2_ TFT devices with Ag as S/D. (**b**) Microscope image of a typical transferred TFT having patterned Ag S/D and patterned MoS_2_. (**c**–**f**) Transfer and (**g**–**j**) output curves of the EHD jet-printed TFTs prepared from 0.025, 0.05, 0.075 and 0.1 M precursor solutions, respectively.

Figure 5a shows a cross-section view of the architecture of printed MoS_2_ TFTs with bottom-gate top-contact structure. The MoS_2_ was either as-grown or transferred on each SiO_2_/Si substrate. Figure 5b shows the top view of transferred TFTs captured by an optical microscope, illustrating the well-defined S/D without wavy edges. Printed MoS_2_ lines were patterned from (NH_4_)_2_MoS_4_ precursor with different concentrations of 0.025–0.1 M to form an active layer with different thicknesses. Ag lines were continuously printed as S/D electrodes with modified Ag paste by the same printing technique at room temperature.

Figure 5c–j show the electrical characterization of transferred TFTs on other SiO_2_/Si substrates prepared from four solution concentrations. In the transfer curves (Fig. 5c–f), all TFTs displayed n-type MoS_2_ behavior. The output curves (Fig. 5g–j) confirmed the typical behavior of MoS_2_. An Ohmic contact property or good linear region was clearly obtained at low V_DS_ in the output curves. The output characteristics show an increasingly saturating behavior for lower MoS_2_ concentration ($\le$ 0.075 M) or thinner thicknesses with V_GS_ < 80 V. The saturated characteristics demonstrated that MoS_2_ channel pinch-off was successfully obtained. When the precursor concentration was enriched up to 0.1 M, the MoS_2_ films showed Ohmic behavior, as indicated in the linear output curve of all values.

The average values of electrical characteristics are summarized in Table 1. Among these concentrations, 0.05 M and 0.075 M exhibited the best electrical properties in printed TFT devices. Indeed, the average linear mobility of 0.05–0.08 cm^2^ V^-1^ s^-1^ and on-to-off current ratio of 10^4^–10^5^ are much higher than those of the 0.025 M ($\sim$ 10^3^) and 0.1 M ($\approx$ 30) cases. The highest value of devices fabricated from 0.075 M was 0.18 cm^2^ V^−1^ s^−1^. It is noted that all the fabricated devices ($\ge$ 20 devices per type of TFT) exhibit similar characteristics, which represents the uniformity and reproducibility of our method.

**Table 1:** Analytical results for electrical properties of transferred MoS_2_ TFTs with the concentration of precursor solution.

(NH_4_)_2_MoS_4_ concentration [M]	I_on_/I_off_	S–S [V dec^−1^]	${\upmu }$_lin_ [cm^2^ V^−1^ s^−1^]
0.025	(1 ± 0.4) × 10^3^	28.4 ± 2.1	0.044 ± 0.002
0.05	(2.3 ± 0.8) × 10^5^	8.47 ± 1.59	0.048 ± 0.007
0.075	(1.0 ± 0.6) × 10^4^	20.5 ± 1.65	0.070 ± 0.010
0.1	(3.0 ± 0.5) × 10^1^	73.1 ± 3.12	0.025 ± 0.002

It is interesting that the non-monotonic relationship of the current ratio, subthreshold slope and mobility with the thickness of MoS_2_ lines varied with the solution concentrations. This behavior can be explained by the resistance network^16^. While the Ag S/D contact the top layer of MoS_2_, access to lower layers involves additional inter layer resistors. The gate electrode mostly impacts the bottom layers of the active layer, and charge screening gives rise to degraded charge carriers for top MoS_2_ layers.

In the thinnest MoS_2_ active layer made with 0.025 M, a lack of sufficient screening of the substrate effect leads to low mobility. Much thicker MoS_2_ layer is obtained from 0.1 M precursor, and the finite interlayer conductivity results in an effectively lower total mobility. The optimal thickness of MoS_2_ was identified as 7–10 layers created from 0.05 to 0.075 M precursors. They possess proper thickness and good surface morphology, which create the highest mobility.

**Figure 6:**
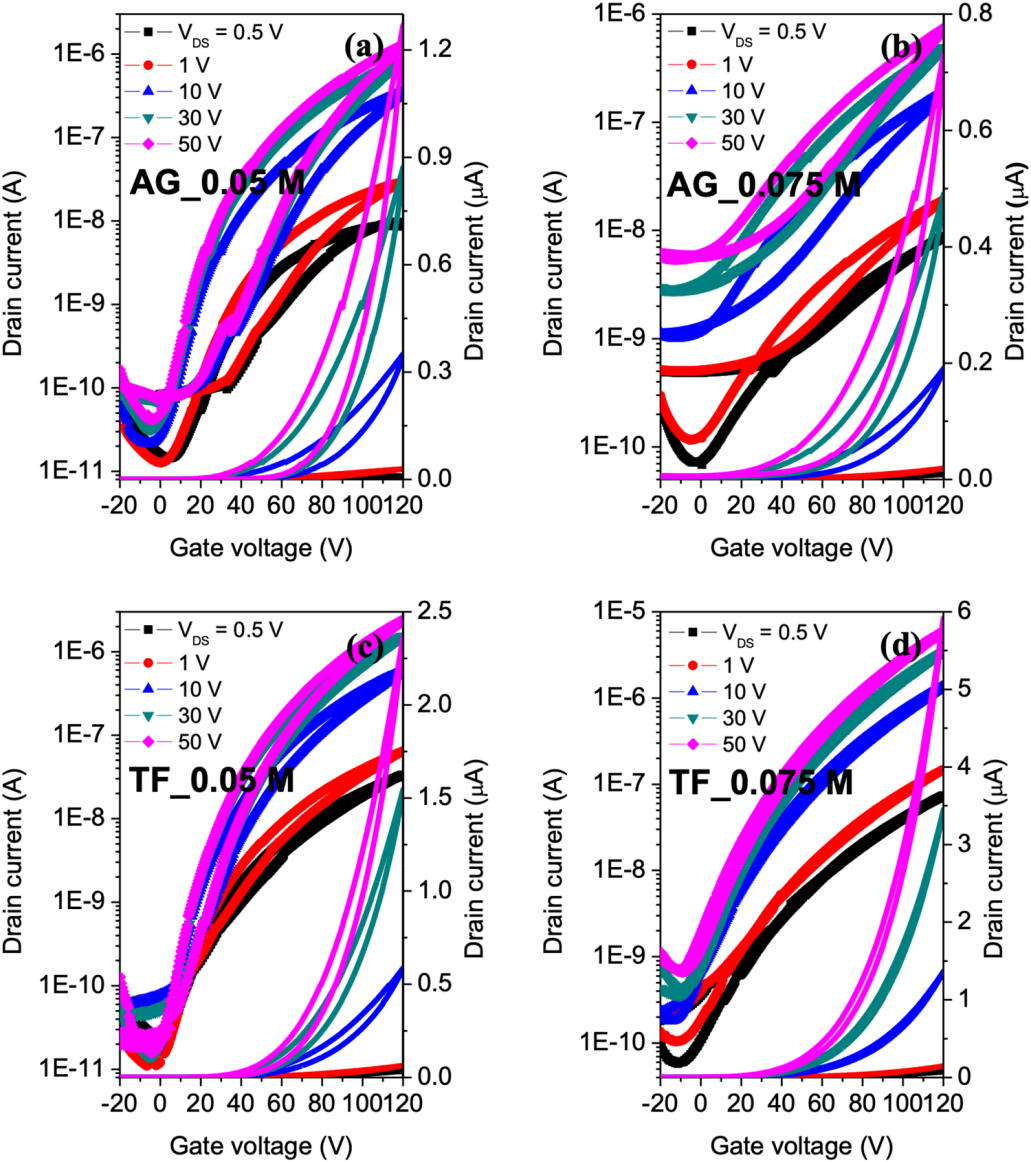
(**a**–**b**) Hysteresis transfer characteristic of TFTs with as-grown (AG) MoS_2_ on SiO_2_/Si and (**c**–**d**) Hysteresis transfer characteristic of TFTs with transferred (TF) MoS_2_ on other SiO_2_/Si prepared form 0.05 and 0.075 M, respectively.

For the hysteresis behavior of TFTs, the relationship of the hysteresis width and the thickness of a MoS_2_ layer was investigated before and after transferring. Figure 6 shows the hysteretic behavior from transfer curves measured at several V_DS_ values for both as-grown and transferred MoS_2_ TFTs. Various thicknesses of the MoS_2_ layer manufactured from precursor concentrations of 0.05 and 0.075 M were chosen to represent all concentrations for TFTs. The whole TFTs show clockwise hysteresis behavior, which can be attributed to carrier traps at the MoS_2_/dielectric interface, and redistribution of charge defects in the dielectric layer.

For more details, the hysteresis of thicker MoS_2_-based devices performed approximately half as well compared to thinner MoS_2_-based TFTs, regardless of transferring MoS_2_. This result agrees well with other work^17^*.* The reasons might come from the redistribution of charges mainly accumulating on the MoS_2_ surface and/or the susceptibility of charge traps at the channel surface to the external electric field owing to the absence of electric screening. One can see the minimum obtained hysteretic gap of 4 V, which is similar to other reported MoS_2_ TFTs with Au/Ti top contacts^18^.

Along with thickness dependence, the hysteresis is found to be related to the transfer of MoS_2_ layer on another substrate in spite of using the same type of dielectric materials. After transferring, we observed 3–4 times smaller hysteresis, 50% decreased subthreshold swing, increased current ratio by 1–2 orders of magnitude, and better mobility for both concentrations, as shown in Table 2. This is clear proof for very good MoS_2_ patterns obtained after transferring by a conventional PMMA based method.

**Table 2:** Analytical results for hysteresis and other performance of TFT devices with concentration of precursor solution and transferring step (AG; as-grown, TF; transferred MoS_2_ films).

(NH_4_)_2_MoS_4_ concentration [M]	MoS_2_	I_on_/I_off_	S–S [V dec^−1^]	${\upmu }$_lin_ [cm^2^ V^−1^ s^-1^]	Hysteretic width [V]
0.05	AG	(3.0 ± 0.5) × 10^4^	15.45 ± 4.36	0.030 ± 0.008	29.30 ± 0.75
	TF	(2.3 ± 0.8) × 10^5^	8.47 ± 1.59	0.048 ± 0.007	9.47 ± 0.56
0.075	AG	(2.5 ± 0.2) × 10^2^	38 ± 3.45	0.005 ± 0.001	14.0 ± 0.25
	TF	(1.0 ± 0.6) × 10^4^	20.5 ± 1.65	0.070 ± 0.010	4.1 ± 0.56

The original SiO_2_/Si used as a substrate to build MoS_2_ might meet undesired contaminants in the ambient environment during the printing process and be affected in annealing to some extent compared to new SiO_2_/Si target substrates. Therefore, owing to the cleaner and undamaged substrate, less scattering and charge carrier trapping might be obtained to promote the performance of fresh SiO_2_-based MoS_2_ TFTs. The hysteresis and the large subthreshold swing are thus explained by interface trap charges present at the MoS_2_/SiO_2_ interface.

The high on–off current ratio of 2.5 × 10^5^, good hysteresis behavior of 4 V, and acceptable subthreshold slope of 6 V dec^−1^ of EHD jet printed MoS_2_ TFTs were comparable to TFTs from other works in terms of current ratio (~ 10^2^–10^4^), such as paper-based MoS_2_ TFTs with printed Cu electrodes^19^, solution-based MoS_2_ FETs with Au S/D^20^, and CVD MoS_2_ TFTs with ink-jet printed Ag or graphene contacts^21,22^. However, the carrier mobility of TFTs was quite modest compared to other MoS_2_ TFTs with different source/drain materials, such as expensive Au/Ti^23^ and so forth. The reason may be poor charge injection from Ag electrodes to the MoS_2_ layer and diffusion of Ag into the underlying layer and channel area. The high contact resistance at the interface between the MoS_2_ active layer and Ag source/drain could be assigned to several factors, such as the high work function of Ag, residual impurities in the original Ag paste or in the printing process, the drying step, and the spatial potential barrier at the MoS_2_–Ag interface. To improve device characteristics, other materials with lower work function could be employed. However, for printed electronics, EHD jet printing technique was used as much as possible to produce our current devices for the purpose of low-cost and environmentally friendly. Consequently, improvement of the interface of the semiconductor and printed Ag electrodes should be focused on the future to optimize device fabrication and circuit integration.

Printed 0.05 M MoS_2_ TFTs had the highest on–off current ratio of 2.3 ± 0.8 × 10^5^ and lowest subthreshold slope of 8.47 ± 1.59 V/dec. TFTs made with 0.075 M showed the highest mobility (0.070 ± 0.010 cm^2^/V s) and the best hysteresis behavior (~ 4 V). Thinner (0.025 M) or thicker MoS_2_ (0.1 M) layers gave rise to degraded TFT properties. The ideal thickness of MoS_2_ for the best performance of TFTs with Ag S/D was 7–10 layers. Although TFTs with an EHD jet-printed active layer and Ag S/D have limited performance, the stacked printed MoS_2_ semiconductor and Ag S/D electrodes using EHD jet printing proved that this printing is a very attractive and effective method for multi-printing technology for 2D materials.

## Conclusions

In summary, MoS_2_ TFTs have been successfully fabricated by EHD jet printing MoS_2_ as a semiconductor and Ag as S/D electrodes for the first time. The MoS_2_-based device exhibits a current ratio of over 10^5^ and acceptable SS slope of 6.0 V dec^−1^, which is important for application in high-speed circuits in the case of SiO_2_ gate insulators. The electrical characteristics of TFTs were observed with different MoS_2_ thicknesses. Higher mobility was found for the most optimal thickness of five to seven layers.

The transferred MoS_2_ TFTs showed better electrical performance compared to as-grown MoS_2_ TFT devices. In addition, after transferring printed MoS_2_, better hysteresis behavior and higher mobility were obtained due to less scattering and charge trapping in the cleaner substrate. The doubled EHD jet printing of the MoS_2_ active layer and Ag S/D electrodes proved the possibility of a low-cost stacked printing method for 2D materials and next-generation optoelectronic applications.

## Methods

### Printing setup

All printing processes for MoS_2_ and Ag were conducted using an EHD jet printer. The system setup included an XY moving stage, a Z motor for three-dimensional movement, a DC power supply, a pneumatic regulator for pressure requirements, a syringe pump, and a nozzle tip with a 100-µm inner diameter for injecting and ejecting (NH_4_)_2_MoS_4_ solution and Ag paste. A voltage was applied from the DC power supply between the nozzle and a conducting stage, which induces an electrostatic field that drives the flow of solution ejected from the nozzle tip, and the flow meets a target. To observe the whole dynamics of the electric field-driven jetting behavior, a high-speed camera was set up to capture or film the process of meniscus deformation and droplet ejection at the tip head.

### Material preparation

The precursor solutions for producing MoS_2_ were synthesized using our recently developed approach^10^. A 1 M sulfur solution was prepared by dissolving S (Alfa Aesar, Fisher Scientific) in carbon disulfide (CS_2_, Yakuri Pure Chemicals Co., Ltd). The precursor solution was obtained by dissolving ammonium tetrathiomolybdate ((NH_4_)_2_MoS_4_, 99.97%, Sigma Aldrich) in 4 parts of ethanolamine (Sigma Aldrich) and 4 parts of butylamine (Sigma Aldrich) with the S solution. Then, 2 parts of n,n dimethylformamide (DMF, Sigma Aldrich) were added to the solution to form the precursor solution. In our CVD-free method, S was added during solution preparation to yield an S-rich precursor instead of adding S powder separately, as proposed in other CVD methods.

The silver paste was formulated by mixing 100 parts of original Ag paste (4000 cps, AD-V7-108) with 1 part of Silveray (solvent) and 3 parts of propylene glycol methyl ether acetate (PGMEA, Sigma Aldrich). This was done to make an even paste and prevent clogging at the tip capillarity during the printing process according to our recent publication^13^. We modified it in order to be relevant to this research.

### MoS_2_ growth and TFT fabrication

Prior to printing, heavily p-doped Si substrates covered by 300-nm thermally grown SiO_2_ were simply cleaned, and 40 min of UV/O_3_ exposure was used to enhance the hydrophilicity. The precursor solution was EHD jet printed on the SiO_2_ with a combination of optimized parameters. The viscosity and surface tension of the precursor solution are around 14 cP and 38 mN m^−1^, respectively. After the printing process, precursor patterns were dried on a hot plate at 150 °C for 30 min and annealed in a furnace tube with an N_2_ environment at 1000 °C for 1 h to form MoS_2_ patterns as a semiconductor layer. MoS_2_ was crystallized through thermolysis of (NH_4_)_2_MoS_4_^24^.

MoS_2_ patterns were then transferred onto another 300-nm SiO_2_/Si substrate for transferred-MoS_2_ TFT fabrication using the conventional wetting method with support of PMMA. Silver paste was subsequently printed transversely on both as-grown and transferred MoS_2_ patterns using the EHD jet printer and dried at 150 °C for 30 min on a hot plate to form S/D electrodes. To obtain a clear channel, printing neat Ag pattern lines was necessary with optimized parameters such as a standoff height of 1.5 mm, substrate temperature of 25 °C, printing speed of 2000 m µ s^−1^, pressure of 80 kPa, and voltage electricity of 1.5 kV. The whole TFT fabrication process is shown in Fig. 1.

### Characterizations

The morphology and thickness of each layer and whole devices were observed by an optical microscope (Olympus-BX51M), atomic force microscope (AFM, Nano expert II EM4SYS), scanning electron microscope (SEM, Tescan, Lyra 3 XMHS) and Raman spectroscopy at a 532-nm excitation wavelength. 2H crystal MoS_2_ structure was measured by transmission electron microscope (TEM, Zeiss Libra 200FE). The electrical characteristics of MoS_2_ TFTs were studied a using semiconductor parameter analyzer in ambient air (Keithley 4200). In order to assess them objectively, a series of at least 20 TFTs for each type was fabricated and characterized.

## Supplementary Information

Supplementary Information.

## Data Availability

The datasets used and/or analyzed during the current study available from the corresponding author on reasonable request.
