# Supplementary material for: Stacked printed MoS2 and Ag electrodes using electrohydrodynamic jet printing for thin-film transistors
Source: Sci Rep. 2022 Dec 28;12:22469. doi: 10.1038/s41598-022-27072-3 (PMC9797473; doi:10.1038/s41598-022-27072-3)
Supplement: Supplementary file 1 — Supplementary Information. [file 41598_2022_27072_MOESM1_ESM.docx]

**Supplementary Information**

**for**

**Stacked printed MoS_2_ and Ag electrodes using electrohydrodynamic jet printing for TFTs**

Thi Thu Thuy Can and Woon-Seop Choi*

School of Electronics and Display Engineering, Hoseo University, Asan, 31499, Korea

**(a)**

**(b)**

**Figure S1.** (a, b) Raman spectrum of MoS_2_ printed from 0.025 M, 0.05 M precursor solution plotted at three points for each concentration.


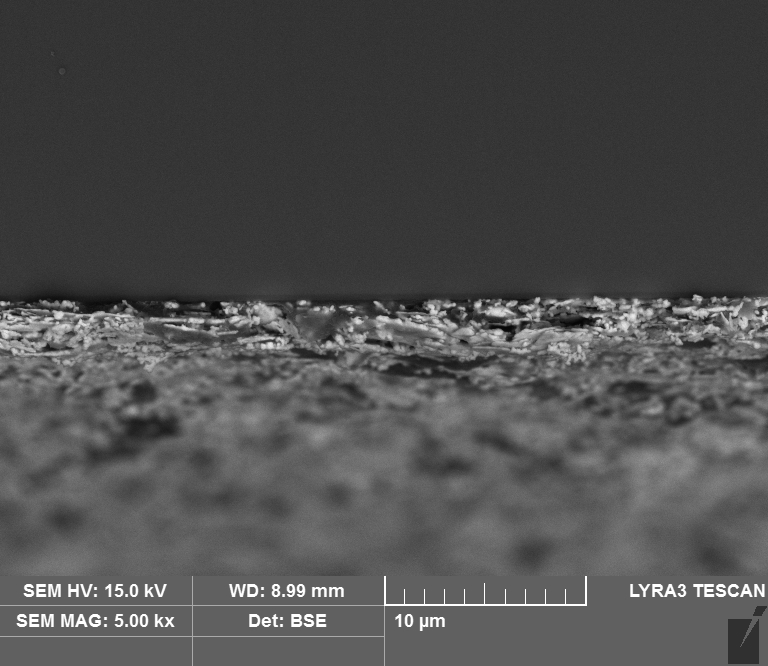


10 $\mu$m


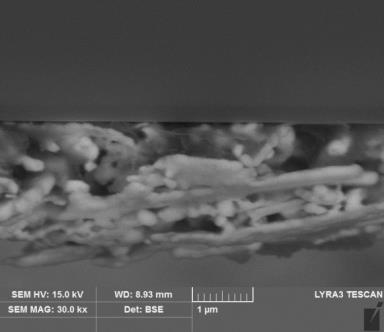


1 $\mu$m

525 $\mu$m Si

300 nm $\mathrm{SiO}_{2}$

2 nm $\mathrm{MoS}_{2}$

2 $\mu$m Ag

**(c)**


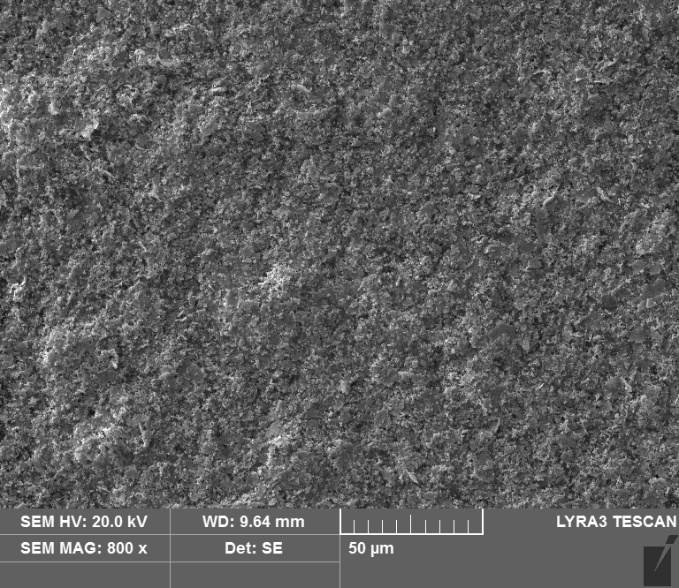


50 $\mu$m

**(a)**


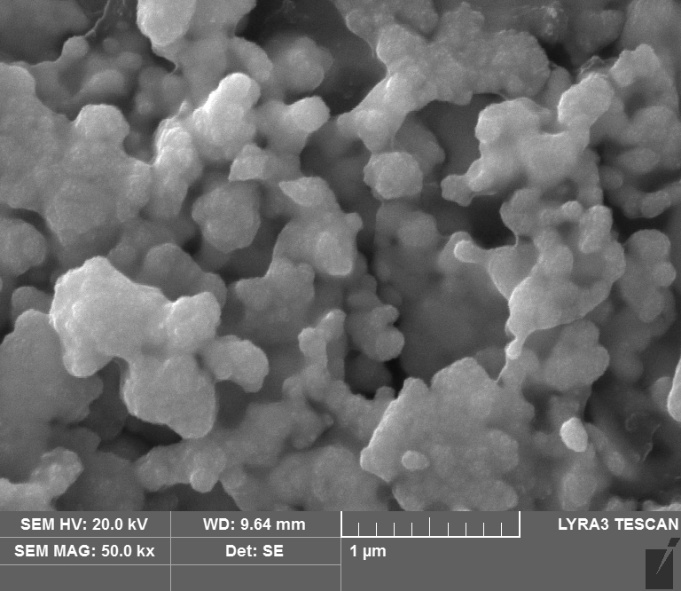


1 $\mu$m

**(b)**

**Figure S2.** Top-view SEM images of EHD jet-printed Ag on MoS_2_/SiO_2_/Si at (a) lower and (b) higher resolution. (c) Cross-section view SEM image of Ag/MoS_2_/SiO_2_/Si. The inset is a higher-resolution image.
